# Gilding the Lily? Enhancing Antenatal Malaria Prevention in HIV-Infected Women

**DOI:** 10.1093/infdis/jix111

**Published:** 2017-02-28

**Authors:** Feiko O. ter Kuile, Steve M. Taylor

**Affiliations:** 1 Department of Clinical Sciences, Liverpool School of Tropical Medicine, Liverpool, United Kingdom; and; 2 Division of Infectious Diseases, Department of Medicine, Duke University School of Medicine, and; 3 Duke Global Health Institute, Duke University, Durham, North Carolina

**(See the major article by Natureeba et al, on pages 29–35.)**

In malaria-endemic Africa, human immunodeficiency virus (HIV) infection and malaria conspire to threaten maternal health: in some regions, nearly 12% of women are coinfected with HIV and malaria parasites during pregnancy [1–4]. Both HIV infection and antenatal malaria individually increase the risks of miscarriage, stillbirth, preterm delivery, and intrauterine growth retardation [1,5]; these effects are amplified because HIV also exacerbates the harmful effects of antenatal malaria for the mother and fetus [5]. In women not infected with HIV, these harmful effects can be partially mitigated by intermittent preventive treatment in pregnancy (IPTp) with sulfadoxine-pyrimethamine (SP), which is broadly recommended throughout sub-Saharan Africa. However, HIV-infected women typically take daily preventive therapy composed of another antifolate combination with antimalarial activity, trimethoprim-sulfamethoxazole (TMP-SMX), which is presumed to provide adequate if not equivalent protection against pregnancy malaria as SP. The evidence base for the efficacy of TMP-SMX is poor [1], and the specific risks that malaria poses to HIV-infected pregnant women suggest the need for enhanced chemoprevention.

A recent multicountry trial involving HIV-infected pregnant women in East and Southern Africa highlighted the limited efficacy of TMP-SMX. Compared with daily TMP-SMX alone, the addition of monthly mefloquine as IPTp in addition to daily TMP-SMX reduced the risks of clinical malaria, malarial infection at delivery, and hospital admissions [6]. Unfortunately, mefloquine prophylaxis was not well tolerated and was also associated with increased maternal HIV load and mother-to-child transmission of HIV [6]. Other options to enhance IPTp have been explored in HIV-uninfected women but have been poorly tolerated or not efficacious and are therefore also unlikely to be suitable for HIV-infected women. These options included IPTp with the fixed-dose combination of chloroquine-azithromycin and IPTp with amodiaquine (alone or combined with SP) [7, 8]. The search for alternative strategies for the prevention of malaria in HIV-infected women remains an urgent priority.

In this issue of *The Journal of Infectious Diseases*, Natureeba et al report the results of the first exploratory trial evaluating the impact in HIV-infected women of IPTp with dihydroartemisinin-piperaquine (DP) [9]. DP is an effective and well-tolerated candidate for IPTp. As an artemisinin-based combination therapy (ACT), DP is currently recommended by the World Health Organization (WHO) for the case management of malaria in the second and third trimesters. A recent comparison of 4 different fixed-dose ACTs for the case-management of malaria in pregnancy showed that DP had the best efficacy and an acceptable safety profile, with an additional benefit of a longer post-treatment prophylactic effect, which supports its suitability as a candidate for IPTp in high transmission areas [10, 11]. This was confirmed in 2 recent exploratory trials in HIV-uninfected women in areas with high SP resistance in Kenya and Uganda, showing that IPTp with DP was much more effective than SP in reducing malaria infection and clinical malaria [12, 13]. Five similar IPTp trials with DP in HIV-uninfected women are ongoing or are scheduled to start soon for further safety assessment and to determine whether this reduction in malarial infection translates into improvements in pregnancy outcome.

Natureeba et al enrolled 200 HIV-infected pregnant women receiving efavirenz (EFV)–based antiretroviral therapy (ART) and using insecticide-treated nets in Uganda into a double-blinded, placebo-controlled trial to compare the effect of daily TMP-SMX alone versus monthly DP when given in addition to daily TMP-SMX. The primary end point was placental malarial infection, assessed by histopathologic analysis (active or past infections). Nearly all women had WHO stage 1 HIV disease and 56% of women had HIV loads below the limit of detection. The study was conducted at the same site as their previous trial with DP as IPTp in HIV-negative women in which, compared with SP as IPTp, DP reduced the risk of this same primary outcome by nearly half [13].

In contrast to the previous trial in HIV-uninfected women, the risk of placental infections in this new trial was very low in both arms, and, compared with that in the TMP-SMX–only arm, the risk was slightly but not significantly higher in the DP plus TMP-SMX arm (3.1% vs 6.1%; *P* = .5). This prevalence of placental infections was approximately 10-fold lower than the risk observed in HIV-infected daily TMP-SMX recipients in the previous years in this same study area [14]. Secondary outcomes determined using loop-mediated isothermal amplification to detect only actively infected placentae came to similar conclusions, with 1 infection detected in the TMP-SMX arm and 3 infections detected in the DP plus TMP-SMX arm. Overall, DP provided no additional benefit for malaria chemoprevention in HIV-infected pregnant women beyond that provided by daily TMP-SMX.

Why did DP confer no additional benefit? First, as the authors note, the low prevalence of placental malarial infection reflects the remarkable drop in malaria transmission observed following the introduction of indoor-residual insecticide spraying in the study district around the time study enrollment began. This effectively undermined the ability to measure differences in efficacy against a rare outcome. In addition, it cannot be excluded that, despite the high levels of parasite cross-resistance to antifolates in the study area, the protection achieved with daily TMP-SMX may be greater than that achieved by IPTp with SP: while SP is taken intermittently starting in the second trimester, often with gaps of several weeks in between courses when reinfections can establish, TMP-SMX is taken daily and also throughout pregnancy, including prior to conception and in the first trimester. This intensive schedule could potentially enhance effectiveness by overcoming partial resistance and by protecting women early in pregnancy, when most new placental infections occur. Last, as the authors note in a recent companion article published elsewhere, the combination of pregnancy and EFV-based ART can potentially induce metabolism of both dihydroartemisinin (by UDP-glucuronosyltransferases) and piperaquine (by CYP isoenzymes) [15]. In this companion article, compared with HIV-uninfected pregnant women, pregnant women receiving EFV-based ART had reductions of 27% in the dihydroartemisinin area under the curve (AUC) 0–8 hours and 38% in the piperaquine AUC 0–21 days [15]. The lower exposure to both dihydroartemisinin and piperaquine suggests that dose adjustments of this fixed-dose combination may be needed in this group. While this current trial used a fixed dose of 3 tablets of DP for all women per the manufacturer’s recommendations, the use of recent weight-based dosage recommendations from the WHO would have rendered a large proportion (approximately 50%) of the women (ie, those weighing ≥60 kg) taking 4 or 5 tablets.

Despite these negative results, the study demonstrates one pivotal finding for putative preventive medications: in women who also received daily EFV-based ART, the addition of monthly DP to daily TMP-SMX appears to be well tolerated. Furthermore, the details provided in the companion article suggested there were no indications that the piperaquine-associated corrected QT prolongations were worse in HIV-infected women receiving TMP-SMX and EFV-based ARTs than in HIV-uninfected women receiving DHA-PIP and were also not associated with pregnancy status or, importantly, with the number of previous IPTp courses taken [15].

Based on this study alone, it would be wrong to conclude that HIV-infected women require no additional antenatal malaria prevention beyond daily TMP-SMX. The power of this study was undermined by the low prevalence of the primary outcome, which was unexpected and beyond the control of the investigators yet undoubtedly beneficial to the participants and their offspring. Encouragingly, the addition to TMP-SMX of monthly DP was well tolerated, which is a necessary precondition for any candidate prevention measure. In HIV-infected mothers, the risk of a mother transmitting HIV to her newborn and of adverse pregnancy outcomes are substantially reduced by using antenatal antiretrovirals. In these women, enhanced antenatal antimalarial efforts will require additional multisite studies with well-tolerated antimalarials to further improve maternal and newborn health. Indeed, incipient studies with monthly DP in HIV-infected women in areas of Kenya and Malawi with higher burdens of malaria should provide compelling efficacy and safety data in the coming years.

## Note

***Potential conflicts of interest.*** F. O. t. K. is a potential recipient of a grant to address the research question discussed in this editorial. S. M. T. certifies no potential conflict of interest. The author has submitted the ICMJE Form for Disclosure of Potential Conflicts of Interest. Conflicts that the editors consider relevant to the content of the manuscript have been disclosed.
